# Molecular Genomic Approaches to Infectious Diseases in Resource-Limited Settings

**DOI:** 10.1371/journal.pmed.1000142

**Published:** 2009-10-26

**Authors:** Josefina Coloma, Eva Harris

**Affiliations:** 1 Division of Infectious Diseases and Vaccinology, School of Public Health, University of California Berkeley, Berkeley, California, United States of America; 2 Sustainable Sciences Institute, San Francisco, California, United States of America

## Abstract

Josefina Coloma and Eva Harris discuss advances in genomics in resource-limited settings and argue that access to training and capacity building in bioinformatics and data mining will be crucial for the future

**Summary Points**

- Researchers in most developing countries lack the technology, resources, and capacity to participate fully in genomics research.
- Information exchange and knowledge translation must be carried out continually through “North–South” collaborations, starting with capacity building in genomics research; “South–South” collaborations must be encouraged to allow countries with limited resources to pool their human and financial capital and share in the benefits of genomics.
- Several emerging countries have made significant progress in the past decade by sequencing the genomes of organisms with little economic value in the developed world but of great local relevance.
- Molecular diagnostics and molecular epidemiology are the first frontier of genomics, with accessible tools that can be applied in resource-limited settings.
- Developing countries entering the genomics era should start by establishing their priorities and enacting appropriate legislation before embarking on large-scale projects.
- Access to training and capacity building of human resources in bioinformatics and data mining are crucial in the developing world.

Only half a century after the landmark discovery of the double helix structure of DNA, the human genome was sequenced and a new era of biomedical research was ushered in [1]. Parallel advances in comparative genomics, genetics, high-throughput biochemical techniques, and bioinformatics have provided researchers in wealthy nations with a repertoire of tools to analyze the sequence and functions of organisms at an unprecedented pace and level of detail. Since the beginning of the genomics era [2],[3], however, it has been evident that researchers in many developing countries will not be participating fully in genomics research, mainly because of their technological isolation and their limited resources and capacity for genomics research combined with the urgency of many other health priorities. To share the benefits of this technology equitably worldwide, some have advocated that developed and developing countries alike should participate in genomics research to prevent widening of the already large gap in global health resources [4]. As most of the funding that has fueled the rapid advance of the field comes from developed country governments, private initiatives, and industry, however, not much has been done to enable poorer countries to participate as equals in genomics research. Developing countries that are not directly participating in a genomics initiative can, nonetheless, gain from the discoveries of this field in a number of ways, as detailed below. It remains to be seen, however, how the developing world will specifically benefit from the refined genetic information and the drugs and vaccines produced as a result of genomics initiatives. Information exchange and translation of knowledge must be carried out continually through fora accessible to researchers in developing countries. “North–South” collaborations—starting with capacity building in genomics research—need to be fostered so that countries that are currently excluded from the genomics revolution find an entry point for participation. “South–South” collaborations must be encouraged to allow countries with limited resources to pool their human and financial capital, learn from each other's experience, and share in the benefits of genomics. Ensuring that the benefits of genomics-based medicine are shared by developing countries involves their inclusion in the discussion of ethical, legal, social, economic, and sovereignty issues (Box 1).

**Box 1. Societal and Ethical Issues in Genomics to Be Discussed with Full Participation of All Nations**

- Issues of confidentiality, stigmatization, discrimination, and misuse of genetic information
- Dangers of a reductionist approach to health issues based only on genetic information that ignores multifactorial determinants
- Issues about intellectual property rights associated with the patentability of DNA sequences, the applications derived from them, and the implications for developing countries [45]
- The potential exploitation of developing-country populations by creating genetic databases for a price [46]
- The potential risk of breeding human beings by design [47]
- Issues about informed consent, standard of care, and availability and pricing of new drugs and vaccines being tested in developing countries [48]

## Initiatives in the Developing World

In the developing world, the link between human genomics and infectious disease is particularly important. The influence of host genes on the differential susceptibility of individuals or populations to infection and the evolutionary influence of pathogens on the genetic composition of populations by selecting for resistant individuals through coevolution can be now dissected in more detail with genomics. An array of host–pathogen interactions are associated with particular human genes and loci, as best illustrated by the relationship of the malaria pathogen with host genetic evolution. As genetic information about larger populations becomes increasingly available, it is important to disseminate information relating genomics to disease as well as to devise intervention strategies for at-risk populations worldwide [5].

Because science and technology are increasingly recognized as vital components for national development, emerging economies and some developing countries are building their infrastructures to promote local innovation and to retain the value of their human, plant, and microbial genomic diversity and research. India, Thailand, South Africa, Indonesia, Brazil, and Mexico, for example, have devoted considerable resources to large-scale population genotyping projects that explore human genetic variation. The Institute for Genomic Medicine (INMEGEN) initiative in Mexico is the largest and most comprehensive, with a broad strategy for incorporating genomics into health care that includes infrastructure, strategic public–private partnerships, research and development in genomics relevant to local health problems, capacity building, and bioethics policy making [6],[7]. Although it is unclear how Mexico will make the transition from early-phase investment to translation of knowledge into products and services with health and economic impacts, the country is taking important steps to address the challenges it and other emerging economies face, such as the shortage of trained professionals and the ability to retain local talent. For example, the National Council for Science and Technology (CONICYT) is making efforts to engage the Mexican scientific diaspora with expertise in genomics by offering repatriation packages tied to jobs at universities and research institutes, an approach that is also being adopted by Brazil.

Brazil's Foundation for Research Support in Sao Paolo (FAPESP) genomics initiative is also considered a political and scientific achievement. Key to its success has been early investment in training young scientists by sponsoring scholarships abroad in areas related to genomics in which Brazil lacks expertise. To avoid brain drain, beneficiaries are required to return to Brazil for at least four years and must have a committed teaching position at a local university before they leave. One important principle of Brazil's genomics initiative is that the projects are relevant to Brazil and the rest of the developing world but are low on the list of priorities of the US and Europe, thus providing both an important contribution to genomics and a benefit to Brazil's economy and scientific endeavor [8]. FAPESP is in the process of sequencing the genes of the parasite that causes schistosomiasis, a disease that afflicts millions in Brazil. Another example in Brazil is the government-funded consortium Organization for Nucleotide Sequencing and Analysis (ONSA), formed to sequence and analyze the genome of the plant pathogen *Xylella*, which infects orange trees and has great economic impact [9]. This effort led to additional genomics projects on vectors of pathogens that cause major public health problems in Brazil, such as the sandfly *Lutzomyia longipalpis*, which transmits *Leishmania* spp., and the *Triatominae* bug species, which are vectors of *Trypanosoma cruzi* [10].

The impact of genomics on the developing world is also illustrated by multinational initiatives such as the one funded by the US National Institutes of Health (NIH), the UK's Wellcome Trust, and private and public institutes in the US and Europe in collaboration with research centers in Brazil, Argentina, Venezuela and Singapore to sequence the genomes of the parasites *T. brucei*, *T. cruzi* and *Leishmania major*, which cause the deadly insect-borne diseases African sleeping sickness, Chagas disease, and leishmaniasis, respectively [11]–[13]. The potential new drug targets identified by these initiatives have great relevance in over 100 developing countries where the diseases take a significant toll on the economy and the quality of life of their citizens. Similar initiatives have resulted in sequencing of other pathogens important to medicine and agriculture. The data from these projects are usually freely available online for data mining and for bioinformatics analysis at remote locations, as most researchers follow the recommendation set by the Bermuda Accord to make DNA sequences (especially human) freely and openly available without delay [14].

Resource-limited countries can enter the genomics era by creating partnerships and regional centers for technology and resources [15]. For example, DNA sequencing technology, still unaffordable for many researchers and public laboratories because of low-use volume and high costs of equipment, reagents, and maintenance, can be affordable if a regional center provides services to a pool of laboratories and researchers within a country or geographical region. As an illustration, using Brazilian infrastructure, Perú and Chile joined the global potato sequencing consortium, which will sequence different varieties of this important agricultural species [16]. Brazil has also generated several open-source bioinformatics tools for the annotation of bacterial and protozoan genomes that can be used by any researcher worldwide [17]. In Africa, the Center for Training in Functional Genomics of Insect Vectors of Human Disease (AFRO VECTGEN) was initiated by TDR (Special Programme in Research and Training in Tropical Diseases) at the World Health Organization (WHO) and the Department of Medical Entomology and Vector Ecology of the Malaria Research and Training Center in Mali to train young scientists in functional genomics who will ultimately use genome sequence data for research on insect vectors of human disease. The program triggers collaborative research with neighboring nations and the vector biology network in Mali, which was built around research grants funded by the US NIH and TDR/WHO [18]. The Malaria Genomic Epidemiology Network (MalariaGEN) uses a consortial approach that brings together researchers from 21 countries to overcome scientific, ethical, and practical challenges to conducting large-scale studies of genomic variation that could assist efforts in the fight against malaria [19]. Successful “North–South” partnerships that help scientists bridge the genomic gap usually involve a project of mutual interest. An example is the common effort of the International Livestock Research Institute (ILRI) in Nairobi and The Institute for Genome Research (TIGR; now the J. Craig Ventner Institute) to sequence and annotate the genome of *Theileria parva*, a cattle parasite that causes important economic losses to small farmers in Africa and elsewhere [20]. This effort has generated local human resources in genomics and infrastructure for the future.

## Application of Molecular, Genetic, and Genomic Tools with Limited Resources

Although the genomics initiatives described above challenge the notion that developing countries must wait to import advances in science and technology that emerge from the developed world, poorer developing countries still do not have the resources to develop their own genomic projects on a large scale. However, implementing simpler molecular genetic approaches to solve health problems is very feasible in resource-limited settings. The decades preceding the human and microbial genome initiatives were highlighted by important developments in molecular and genetic methods applied to infectious diseases. These developments were enabled by increasingly available genetic information about many pathogens and their vectors and by molecular tools such as PCR and powerful sequencing technologies, which permitted rapid advances that were successfully introduced into the developing world with little delay.

Molecular tools for diagnosis have gained a ready foothold because many poor countries do not have the facilities for traditional diagnosis and surveillance. Thus, diagnosis often relies on clinical observations or requires that a sample be sent out to foreign agencies such as the US Centers for Disease Control and Prevention (CDC) for confirmation. In addition, even when available, classic techniques based on serological, microscopic, and culture-based methods are often lengthy, of only moderate sensitivity, and not highly discriminatory at the level of species subtype or strain. By adapting DNA technologies to the existing infrastructure, using home-grown solutions to reduce their cost, and applying them to solve local health problems, molecular approaches to detect and type infectious agents on-site offer real value [21]. Fostering appropriate technology transfer and capacity-building in the “South” enables public health laboratories and research groups in less scientifically developed countries to participate in global genomics by contributing their findings and sharing their expertise with their peers [22],[23]. For example, we and others adapted PCR-based molecular diagnostic techniques for infectious diseases such as leishmaniasis and dengue for cost-effective application in laboratories with minimum infrastructure and basic technical expertise, which are now fully validated and used routinely throughout Latin America [21],[24]–[30]. This approach relies on understanding the principles of the technologies, deconstructing them into their basic components, and rebuilding them on-site [21].

Another area where molecular tools have demonstrated their utility in resource-poor settings is in detecting drug resistance in a variety of pathogens. This has been facilitated in large part by successful “North–South” partnerships that have served to train scientists in developing countries in the use, implementation, and interpretation of modern molecular methods applied to emerging drug resistance (see [31]). This approach has been particularly successful with certain diseases, such as malaria, HIV/AIDS, tuberculosis, and drug-resistant bacterial infections (both nosocomial and community-based). Unfortunately, most studies of drug-resistant pathogens are performed independently of one another, so data on the prevalence of resistance markers is scattered in disparate databases or in unpublished studies without links to clinical, laboratory, and pharmacokinetic data needed to relate the genetic information to relevant phenotypes. To enable molecular markers of malaria drug resistance to realize their potential as public health tools, the Worldwide Malaria Resistance Network (WARN) database is being created with the dual goals of improving treatment of malaria by informed drug selection and use and providing a prompt warning when treatment protocols need to be changed [32],[33]. By accelerating the identification and validation of markers for resistance to combination therapies, this global database should help prolong the useful therapeutic lives of important new drugs.

The ultimate power of genetic tools in resource-limited settings is evident in the field of molecular epidemiology, where genetic information about the host or infectious agent is analyzed together with clinical and epidemiological data to derive and implement appropriate interventions. For example, molecular tools based on limited sequence information, such as molecular fingerprinting of a polymorphic marker, have made important contributions to strengthening control of tuberculosis in both developed and developing countries by enabling analysis of transmission patterns, helping identify phenotypic variation among strains, and facilitating evaluation of the global distribution, relative transmissibility, virulence, and immunogenicity of different lineages of *M. tuberculosis* [34]–[38]. Bacterial infections, food-borne outbreaks, and viral infections in developing countries, including the recent H1N1 influenza pandemic, are monitored using similar typing methodologies [39]–[41]. Molecular tools permit a refined case definition and thus have tremendous potential for decision-making support and informing targeted public health interventions in countries with high burdens of disease and limited technological capabilities and resources.

The trend to move beyond genetic marker analysis to full genome sequencing is growing, as complete genome data can provide a wealth of information about etiologic agents of disease that was previously unknown. Full-genome approaches are not always necessary, however. In molecular epidemiology of infectious diseases, nucleic acid fingerprinting can provide enough answers to important epidemiological questions to allow critical interventions to be designed (see above). In fact, too much genetic information, in some instances, can obscure the picture, as several closely related pathogenic variants might coexist in one individual or one outbreak that differ by only a few nucleotides but that nonetheless belong to the same strain or subtype, complicating the interpretation of results [42].

The relatively rapid transfer of DNA technology from developed to developing countries is an excellent example of what can be done by forging strong relationships between universities and research groups and public-health laboratories across the world. The validity of adapting these technologies relies on links with epidemiological data and translation into local public health interventions.

## Setting Priorities

General international ethical and scientific guidelines for genomics have been created and are being adapted by nations participating in the field as it evolves. Governments and regulatory agencies in the “North” have prepared for the eventual implementation of genomics-based medicine in their respective countries. A critical problem faced by developing countries is the lack of national guidelines for genomics research and its ethical ramifications. Thus, a priority to be set by countries in the early steps of genomic applications is to draw up the necessary rules and legislation on genomics and to generate procedures for implementation. Creating the necessary communication channels between researchers, social scientists, policy makers, and civil society organizations is also a critical step. Other key challenges facing emerging genomics researchers include proper informed consent and privacy protocols for research participants, protecting them against the potential discrimination that might emerge from genetic information and ensuring that any benefit that comes to fruition from the research reaches them. In parallel, capacity building of scientists in clinical research and of ethics committees in these issues is essential. Past experience with “safari research” in which biological samples are taken out-of-country for research that does not benefit local populations have prompted countries such as Mexico, India, and Brazil to draw up legislation governing “sovereignty” over genomics material and data that restricts the export of biological materials for studies abroad and prioritizes national interests. Poorer countries currently lacking their own genomics initiatives could benefit from similar legislation balancing the protection of “genomic sovereignty” while fostering international collaborations that bring much-needed resources and increase local scientific capacity. Beyond the improvement of their basic genomics research capabilities, governments should engage their relevant ministries to develop a plan to integrate genetic and genomics products (including diagnostics, vaccines, therapies, and others), within the health system and public health programs with emphasis on accessibility and equity to improve health for all. A good example of priority setting in genomics is Mexico's national genomics program over the last 15 years (see Box 2).

**Box 2. Building a Road toward Genomics: The Mexican Experience 1995–2009 [7]**

- Increases in investment in science and technology (S&T) from 0.35% to 0.43% of the GNP and creation of national S&T legislation to increase regional funding
- Four-fold increase in number of students registered for doctoral-level programs
- Participation in international genomics efforts
- Creation of sequencing initiatives of organisms with local agricultural and health relevance
- Creation of a Genomics Sciences degree and two scientific societies in genomics
- Creation of the National Institute of Genetic Medicine (2004-INMEGEN) with seed funding for modern infrastructure; a strategy for development that includes country-wide strategic alliances; high-level research and academic programs; ethical, legal, and social implications of genomic medicine; and translation of the scientific knowledge into public goods
- Establishment of genomics research priorities based on most prevalent local diseases
- Plans for creation of public–private partnerships to guarantee sustainability

## Sharing Know-How

**Figure 1:**
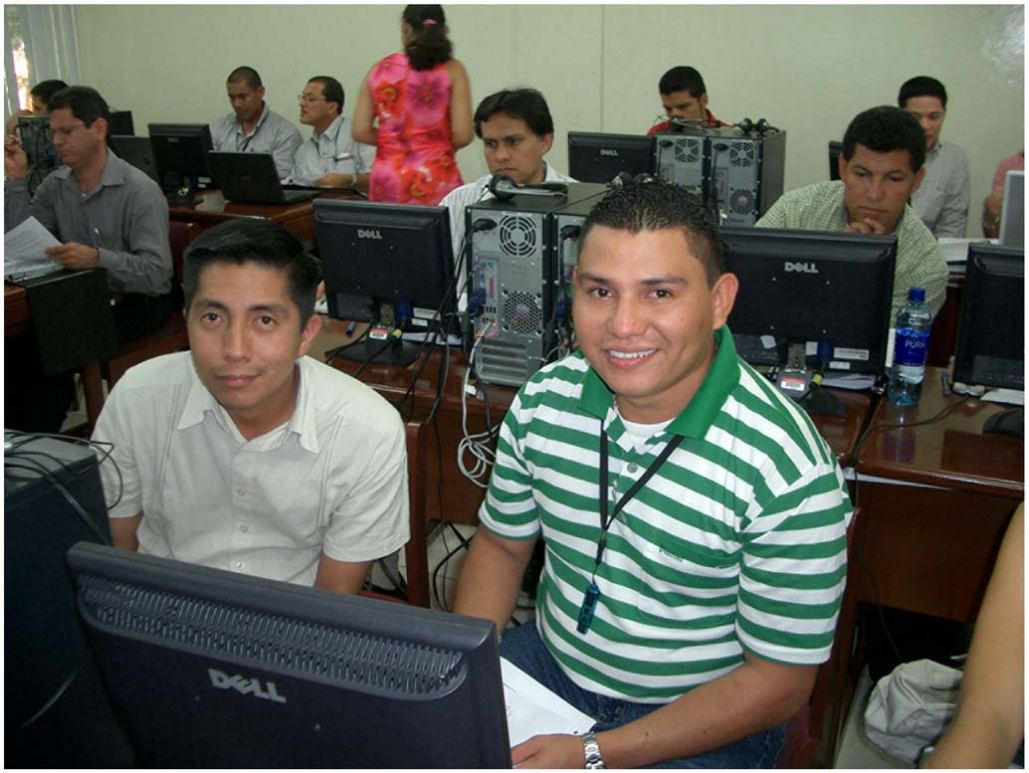
Participants in a Bioinformatics/Genomics Analysis workshop in Managua, Nicaragua, in June 2008 (conducted by the Sustainable Sciences Institute and the Broad Institute). Photograph by Eva Harris.

To strengthen genomics globally, the tools necessary for analysis of genomics data are urgently needed in developing countries, where they are currently underutilized [43]. A problem with genomics is that much of the advanced knowledge is concentrated in individuals and a few research centers and companies rather than in textbooks or academia, restricting dissemination even though massive amounts of genomic data and software are openly accessible through the Internet. A conscious effort on the part of developed nations to transfer their knowledge of the use and analysis of genomic databases needs to be encouraged to help developing countries manage their own specific data on indigenous biological species, local epidemiology and infectious diseases, biodiversity, and other issues. Some successful programs and initiatives include the Wellcome Trust Sanger Institute training courses on bioinformatics and genomic analysis, the Sustainable Sciences Institute–Broad Institute bioinformatics workshops (Figure 1), and the TDR/WHO-South African Bioinformatics Institute (SANBI) regional training center. Online training like the S-star alliance bioinformatics courses and conferences such as the African Bioinformatics Conference (Afbix'09) with remote participation are becoming more widespread and are an excellent option for countries with limited resources. GARSA (Genomic Analysis Resources for Sequence Annotation) is a flexible Web-based system designed to analyze genomic data in the context of a data analysis pipeline. Hosted in Brazil, this free system aims to facilitate the analysis, integration, and presentation of genomic information, concatenating several bioinformatics tools and sequence databases with a simple user interface [44]. An alternative to on-site sequencing is to partner with colleagues in more-developed countries to have samples processed abroad in sequencing centers. This is possible only if local legislation allows for export of biological samples, and if true partnership and trust exist with a colleague(s) in the developed country.

## Challenges for the Future

As developing countries reevaluate their role in the genomics era, they will continue to explore the unique opportunities that arise from the vast natural and genomic diversity that they embody. As exemplified by the successes in Brazil, Mexico, and several African countries, it is possible to turn challenges and problems such as emerging and endemic infectious diseases into opportunities for unique scientific and economic growth. Access to sequencing facilities, open-source databases, and harmonized methodologies for genomic analysis are essential for the future of genomics in the developing world. However, unless a more concerted effort is made to include countries with limited scientific development and resources, it is unlikely that they will fully participate in genomics projects or use the technologies available other than by allowing their genetic material to be accessible to others. As emerging countries set their own priorities for genomics research and take ownership of its results, the main challenge across developing nations remains access to training and knowledge translation. Human resources and local capacity in genomics are thus central to development, as countries with these skills could participate in the potential benefits of the field with respect to health, food security, natural resource management, and other critical areas. “North–South” and “South–South” collaborations are a viable and extremely rewarding way to increase the capacities of developing countries to access genomic tools to address unique problems considered of little economic value outside these countries but of tremendous importance to the majority of the world's population.
